# Supplementary material for: Graphene Materials from Coke-like Wastes as Proactive Support of Nickel–Iron Electro-Catalysts for Water Splitting
Source: Molecules. 2024 Mar 20;29(6):1391. doi: 10.3390/molecules29061391 (PMC10975166; doi:10.3390/molecules29061391)
Supplement: Supplementary file 1 [file molecules-29-01391-s001.zip › molecules-2918790-supplementary.pdf]

Supplementary Materials

Graphene materials from coke-like wastes as proactive support of nickel–iron electro-catalysts for water splitting

María González-Ingelmo[1], Victoria G. Rocha[1], Zoraida González[1], Uriel Sierra[2], Enrique Diaz Barriga[2], Patricia Álvarez[1].*

1 Instituto de Ciencia y Tecnología del Carbono (INCAR), CSIC, Francisco Pintado, Fe 26, 33011 Oviedo, Spain;

2 Laboratorio Nacional de Materiales Grafénicos, Centro de Investigación en Química Aplicada, Blvd. Enrique Reyna Hermosillo, 140, Saltillo 25294, Mexico;

* Correspondence: par@incar.csic.es (P.Á.); Tel.: +34-985119090 (P.Á.)

1. SEM-EDX characterization of TCP-GO-G-NiFe

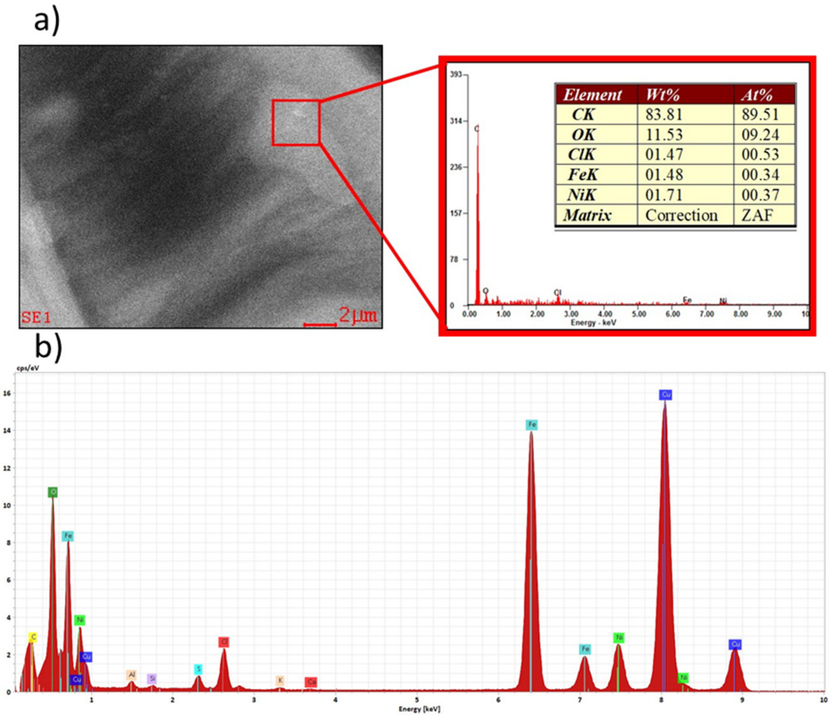

Figure S1: (a) SEM-EDX spectrum of TCP-GO-G-NiFe. (b) TEM-EDX of TCP-GO-IW-NiFe.

2. STEM-EDX analysis of the 3D electrodes

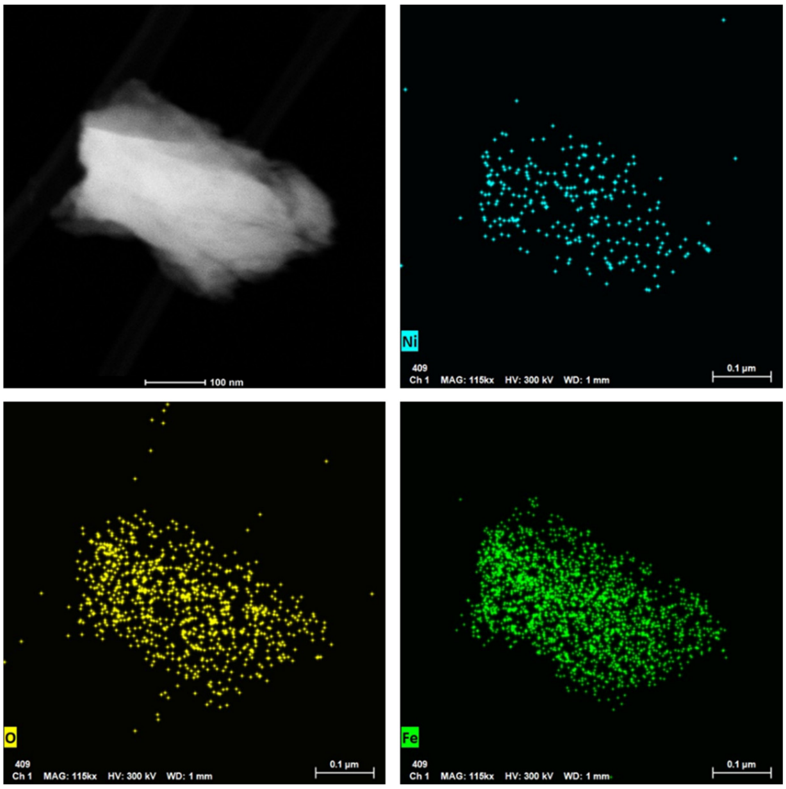

Figure S2. STEM-EDX images and mapping of TCP-GO-G-NiFe.

3. Graphene oxide preparation

The chemical oxidation of graphite-to-graphite oxide makes use of the Hummer's reagents and additional amounts of $NaNO_3$ and $KMnO_4$. A total of 360 mL of concentrated H_2SO_4 was slowly added for a period of 2h to a flask containing the raw carbon-based material (7.5 g) and $NaNO_3$ (7.5 g). Once the mixture was homogeneous and stable, 45 g of $KMnO_4$ was added in small doses, and an ice bath was used to keep the temperature below 20 °C. The solution was then heated at 35 °C and stirred for 8 h. After this time, 1.5 L of 3% H_2O_2 was slowly added, and the mixture was stirred for an additional 30 minutes. The mixture was centrifugated at 3700 rpm for 30 min, and the obtained solid was washed with deionized water and centrifugated again until neutral pH was reached. Then, the aqueous suspension obtained was sonicated for 8 hours in an ultrasound bath. The obtained samples were labeled as GO-IW (from the coke-like waste) and GO-G when prepared from standard graphite.

4. Electrochemical measurements

The cell configuration included the previously prepared 3D electrodes as the WEs, an Ag/AgCl/3.5 M KCl as RE, and a graphite rod serving as the CE. The electrochemical behavior of the samples was evaluated in the potential range between 1.2 and 1.8 V vs. reversible hydrogen electrode (RHE). Potentials in this section are converted to the RHE scale for comparative purposes and are calculated according to the Nernst equation: $E_{RHE} = E_{Ag/AgCl} + 0.059pH + 0.216$ V. Initially, cyclic voltammetry (CV) experiments (50 mVs^{-1}) were performed to stabilize the OER activity of the active materials. A linear sweep voltammetry (LSV), at 10 mVs^{-1}, was then conducted, and the corresponding Tafel slope values were calculated. The catalytic activity is reported in terms of current density in $mAcm^{-2}$, where cm^2 is the geometric exposed area of the WE ($1cm^2$). The stability of the electrodes TCP-GO-r-NiFe was assessed through chronopotentiometry (CP) experiments at 10 $mAcm^{-2}$ for 1 hour. Electrochemical impedance measurements (EIS) were also carried out on these electrodes to evaluate the R_{ct} values. In the EIS experiments, a potential perturbation of 10 mV was applied within a frequency range spanning from 100 kHz to 100 mHz. The EIS measurements were acquired under a constant potential of 1.6 V vs. RHE, potential at which OER is assumed to occur. The electrochemical measurements were performed on a BioLogic VMP Multichannel Potentiostat.

5. Hand-made catalytic cell configuration

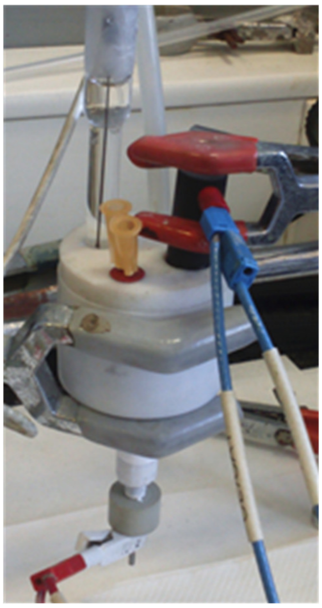

Figure S3. Home-made three-electrode cell set up for the electrocatalytic tests.

6. XPS general spectra of TCP-GO-G-NiFe and TCP-GO-IW-NiFe

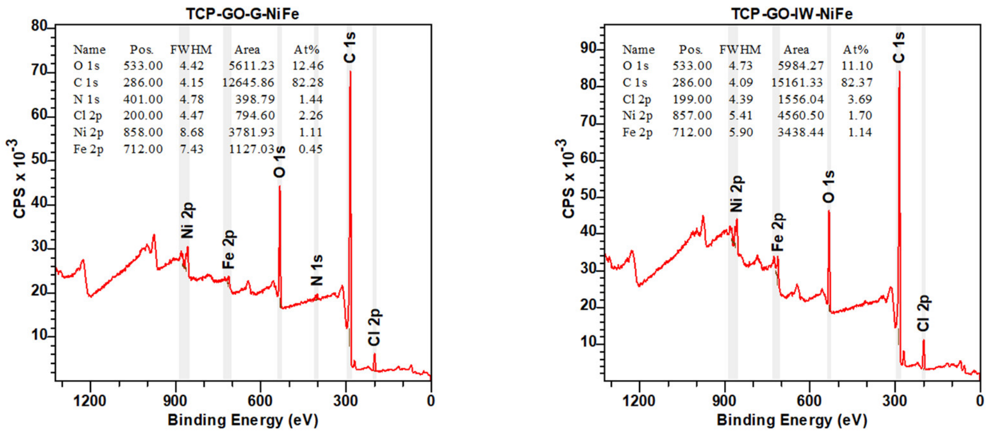

Figure S4: XPS general spectra of TCP-GO-G-NiFe (left) and TCP-GO-IW-NiFe (right).

7. Catalytic results

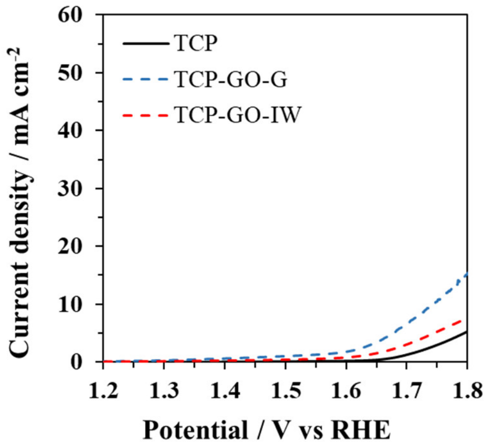

Figure S5: Linear sweep voltammetry of the bare electrodes prior NiFe electrodeposition. Experiments were carried out a 10 mVs^{-1} using N_2-saturated KOH 1 M as electrolyte.

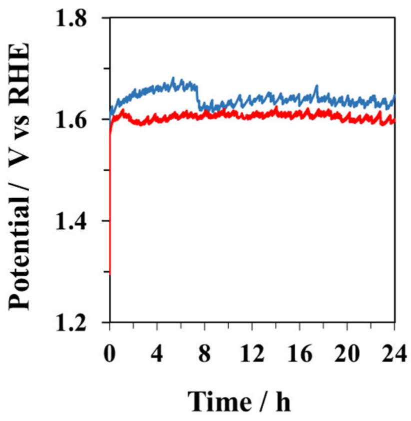

Figure S6: Long-term stability test. Chronopotentiometry experiments of the samples TCP-GO-G-NiFe (blue) and TCP-GO-IW-NiFe (red) recorded at 10 mAcm^{-2} for 24 hours in N_2-saturated 1 M KOH.

8. Raman characterization of graphene oxides GO-G and GO-IW

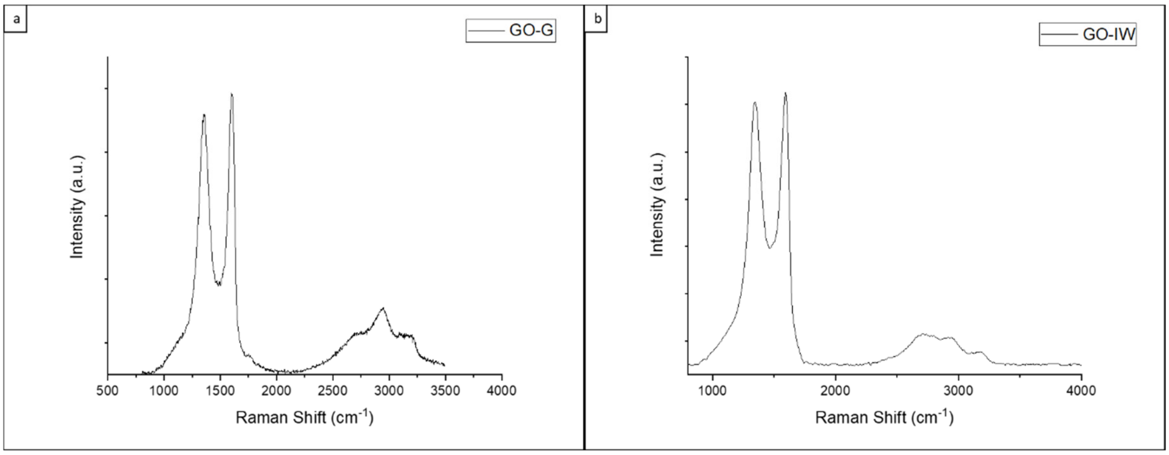

Figure S7: Raman spectra of (a) GO-G and (b) GO-IW.
